# The Simulation of In-Situ Groundwater Detector Response as a Means of Identifying Beta Emitting Radionuclides by Linear Regression Analysis

**DOI:** 10.3390/s21175732

**Published:** 2021-08-25

**Authors:** Graeme Turkington, Kelum A. A. Gamage, James Graham

**Affiliations:** 1 Electronics & Electrical Engineering, School of Engineering, University of Glasgow, Glasgow G12 8QQ, UK; g.turkington.1@research.gla.ac.uk; 2 National Nuclear Laboratory, Sellafield, Seascale, Cumbria CA20 1PG, UK; james.graham@uknnl.co.uk

**Keywords:** strontium, beta, radiation, detectors, linear, regression

## Abstract

The in-situ characterisation of strontium-90 contamination of groundwater at nuclear decommissioning sites would represent a novel and cost-saving technology for the nuclear industry. However, beta particles are emitted over a continuous spectrum and it is difficult identify radionuclides due to the overlap of their spectra and the lack of characteristic features. This can be resolved by using predictive modelling to perform a maximum-likelihood estimation of the radionuclides present in a beta spectrum obtained with a semiconductor detector. This is achieved using a linear least squares linear regression and relating experimental data with simulated detector response data. In this case, by simulating a groundwater borehole scenario and the deployment of a cadmium telluride detector within it, it is demonstrated that it is possible to identify the presence of ${}^{90}$Sr, ${}^{90}$Y, ${}^{137}$Cs and ${}^{235}$U decay. It is determined that the optimal thickness of the CdTe detector for this technique is in the range of 0.1 to 1 mm. The influence of suspended solids in the groundwater is also investigated. The average and maximum concentrations of suspended particles found at Sellafield do not significantly deteriorate the results. It is found that applying the linear regression over two energy windows improves the estimate of ${}^{90}$Sr activity in a mixed groundwater source. These results provide validation for the ability of in-situ detectors to determine the activity of ${}^{90}$Sr in groundwater in a timely and cost-effective manner.

## 1. Introduction

This research aims to develop a methodology for estimating the ${}^{90}$Sr activity in contaminated groundwater at nuclear decommissioning sites as measured by a cadmium telluride (CdTe) [1,2] detector that is deployed in a groundwater borehole. There is demand in the nuclear decommissioning industry to develop new ${}^{90}$Sr monitoring techniques that can reduce the lifetime monitoring costs of the radionuclide [3,4,5,6,7,8]. The aim is to design techniques that are safer, produce less secondary waste and can be completed rapidly without the need for specialised laboratory techniques. Currently, groundwater samples are taken from underground boreholes, treated, placed into storage and, eventually, the radionuclides in the groundwater are analysed using radiochemical separation and liquid scintillation counting. However, these stages could be bypassed with the deployment of an in-situ semiconductor detector. In order to compete with existing techniques, an in-situ detector should be able to identify the activity of individual radionuclides. This paper reports on a technique that allows for the activity of individual radionuclides to be determined using a gross measurement of the activity in the borehole and predictive modelling. Each radionuclide’s contribution to the total activity is estimated by relating the gross spectrum to the simulated detector response to each radionuclide in the scenario.

There are two main challenges to consider when designing a beta detector. The first is that beta particles have a limited range in matter and are easily attenuated as they travel through the environment [9]. The consequence of this is that the detection medium must be placed very close to the source of the radiation [10]. This is the reason that liquid scintillation is one of the primary beta detection techniques [11]. By mixing the sample of radionuclides and the scintillator together, the likelihood of interaction between beta radiation and the scintillator is sufficiently high. Liquid scintillation counters are able to achieve a low limit of detection, below the guideline value for drinking water. Alternatively, this research has taken advantage of the development of high-quality, high-density and high-energy-resolution semiconductors to design a compact detector that can be immersed directly in contaminated groundwater. Using a thin layer of waterproofing, the detector can be deployed in contaminated groundwater while keeping the attenuation of the radiation spectrum minimal.

The other challenge is that beta decay results in particles that are emitted over continuous spectra and terminate at an end-point energy. This presents difficulties when identifying beta emitters from a mixed source of radiation. When ${}^{90}$Sr decays via beta emission, it is a result of an excess of protons in the nucleus, one of which will convert into a neutron, a beta particle and an anti-neutrino. The emission of the anti-neutrino during beta decay is significant as the energy released by the nuclear decay is shared between both particles, resulting in the continuous spectrum of emission. As a result, it is difficult to determine the origin of a particular beta particle in a mixed spectrum and, consequently, the radionuclides in the sample. By contrast, gamma emission occurs as electrons fall between fixed energy levels in the nucleus [12]. This results in characteristic energy peaks in the emission spectra, from which the associated radionuclides can be easily identified. As it is not practical to perform radiochemical separation in situ, the detection of ${}^{90}$Sr will require the acquisition of a gross beta spectrum and predictive model to identify the constituent radionuclides. This method posits that the total spectrum measured with the detector comprises the summation of the detector response to each individual radionuclide. By simulating the detector response to the constituent radionuclides, a linear regression model can be applied to make a maximum-likelihood estimate of the radionuclides present in the groundwater and their activities.

### Background Literature

Predictive modelling techniques have been developed for applications with different detector types. A simulation-based linear regression approach to radionuclide identification has been demonstrated in other research [13,14]. S. Grujic et al. [15] report on using the Monte Carlo simulation package MCNP [16] to simulate the response function of a semiconductor Si detector to identify ${}^{90}$Sr contamination in water samples taken from spent nuclear fuel storage pools. In this case, a PIPS (passivated implanted planar silicon) detector with a surface area of 1.197 cm and a thickness of 502 µm is simulated. In this research, the gross radiation spectrum is considered to be the sum of the individual radiation spectra, modified by the response function of the detector—as described in Equation (1).

(1) $$\sum_{n=1}^{N}A_{n}R_{n,i}=C_{i}i=i_{L},i_{U}$$

where *i* is the lower and upper channel number of the multichannel analyser in the detector, *N* is the number of unknown radionuclides with unknown activities in the sample, $A_{n}$ is the activity of the radionuclide that must be determined [Bq], $R_{n,i}$ is the simulated response function of the detector to radionuclide *n* in channel *i*, and $C_{i}$ is the gross beta spectrum that is measured with the detector in the configuration as modelled in the simulation. This equation can be used to form a system of linear equations that are resolved using the least squares with linear inequality constraints method [17]. In this study, the groundwater sample was placed in a Petri dish below the detector and the scenario was modelled in MCNP. The researchers used the least squares method to identify an activity of 166.3 ± 13.4 Bq/cm${}^{3}$ for ${}^{137}$Cs in a mixed sample with ${}^{90}$Sr. The activity of ${}^{137}$Cs in the sample was found to be 179.8 ± 10.5 Bq/cm${}^{3}$ using a calibrated GX5020 germanium (Ge) detector.

E. Bai et al. conducted an investigation into the use of portable gamma detectors for identifying radionuclides in public spaces and at checkpoint searches with the goal of identifying nuclear weapons. In this case, the researchers were interested in poorly resolved gamma spectra from unknown radionuclides, where there may be potential overlap of gamma emission or sufficient attenuation of their characteristic peaks to mask their presence. They proposed a technique where the detector repsonse is modelled, but they presented a two-stage algorithm consisting of a linear regression stage and a subsampling stage based on majority voting [18]. The detection algorithm is based on a similar equation to Equation (1). In typical gamma detection, a peak fitting algorithm is used to fit a Gaussian function to peaks in detected gamma spectrum, and the energies of these peaks are used to identify the radionuclides from a database of radionuclides. The scenario that the researchers address is when the gamma peaks are masked by noise or so poorly resolved that peak fitting may fail. The generated system of equations was resolved using a least absolute shrinkage and selection operator (LASSO) algorithm with a subsampling stage implemented to successfully reduce the rate of false positive errors. Predictive modelling is increasingly being used in other fields to overcome the deficiencies in detector technology as a means to implement remote sensors for monitoring environmental quality [19,20,21,22].

## 2. Methodology

The radionuclide identification procedure presented in this research comprises two sections. First, the detector response is simulated [23], and second, a system of equations is constructed that relates the detector response to individual radionuclides to the gross radiation spectrum that is measured. A linear regression is applied to the system of equations to provide an estimate of the activity of the radionuclides in the groundwater.

### 2.1. Groundwater Radiation Model

The gross radiation spectrum collected with an in-situ detector comprises contributions from each individual radionuclide present in the groundwater. This can be expressed by a linear relationship, as written in the form of Equation (2).

(2) M=RN

**M** is a vector containing the gross spectrum of radiation measured with a CdTe detector deployed in a groundwater borehole. **N** is an n×k matrix that details the detector response to each individual radionuclide, where *k* is the number of radioactive sources and *n* is the number of channels in the detector. The relative activities of the radionuclides, R, are unknown and can be found by solving Equation (2) computationally using linear regression [24]. In this case, the iteratively weighted least squares method is used [25].

### 2.2. Detector Response Simulation

In nuclear radiation spectroscopy, the spectrum that is output by a detector is an approximation of the true emission spectrum released by radionuclides. These two spectra can be related by a response function. There are two aspects that contribute to the response function. Firstly, the radiation must interact physically with the environment en route to the detector and this leads to attenuation of the spectrum. This process is simulated using the Monte Carlo software, Geant4 [26]. Secondly, the contribution of electronic noise and statistical variations in electron-hole production [12] within the semiconductor must be modelled, and this is achieved with a Gaussian broadening of the detector output generated by Geant4. This is expressed in Equation (3) [27].

(3) $$\int_{0}^{E_{min}}R\left(S,E\right)N\left(E\right)dE=M\left(S\right)$$

(4) $$0\leq E\leq E_{max},0\leq S\leq S_{max}$$

In this equation, *N*(*E*) is the emission spectrum, *M*(*S*) is the measured radiation spectrum, *R*(*S*, *E*) is the response function, *S* is the signal value, and *E* is the radiation energy. This equation has the form of a Fredholm integral equation and can be rewritten as a system of linear equations; see Equation (2).

**Figure 1:**
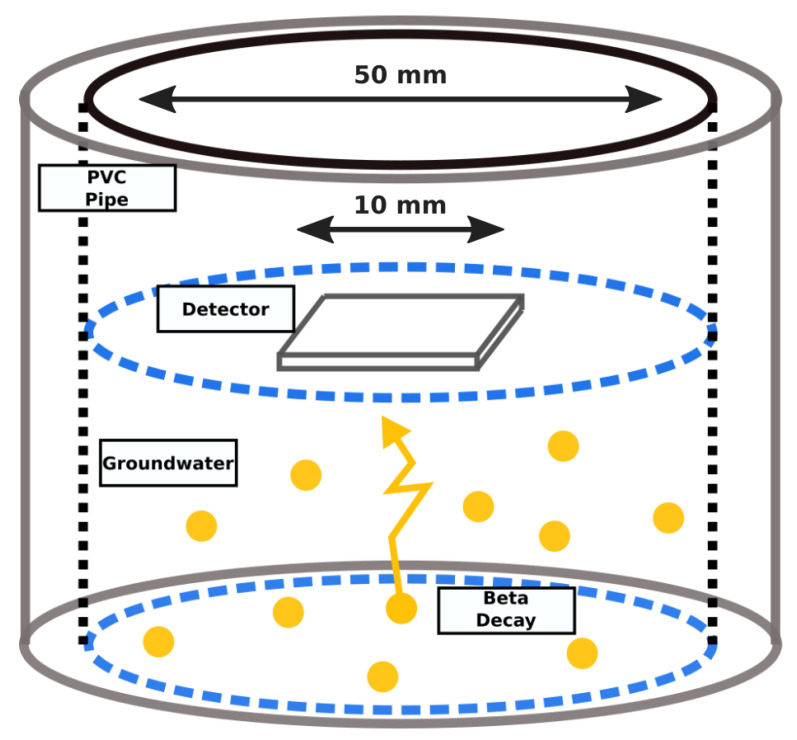
The scenario simulated in the simulation. A PVC pipe, with a 5 cm diameter, comprises the innermost layer of the borehole and it is surrounded by silicon. The detector is deployed in contact with the water, which is populated randomly with decaying radionuclides.

The groundwater borehole environment in which the detector will be deployed was modelled with a Geant4 simulation; see Figure 1. The purpose of the simulation is to model the decay of radionuclides and the interaction of the released radiation with the environment and the detector. A typical groundwater borehole at Sellafield comprises a plastic inner layer with 5 cm diameter and is surrounded by a silicon filtering layer, which is installed to keep contaminants out of groundwater samples. The interior of the borehole was filled with water to a depth of 5 cm, with the detector positioned at the surface.

The detector design consists of a 10 × 10 × 1 mm CdTe detector with 20 nm thick platinum Ohmic contacts on either side. The detector is sealed with two layers of low-density polyethelyne (LDPE), a thin and waterproof plastic, which protects the detector and electronics from the groundwater but also attenuates incoming beta particles. There is a 1 mm gap between the detector and the LDPE that is filled with air.

To generate data to fill the response matrix, **N**, the groundwater was populated with the equivalent of 100,000 BqL${}^{-1}$ ${}^{90}$Sr, ${}^{90}$Y, ${}^{137}$Cs, ${}^{40}$K for 5 h of measurement in the borehole. This simulation used the FTFP BERT physics list, which allows electron interactions up to 100 TeV and uses a maximum step length of 0.65 mm, an energy threshold of 900 keV and a low energy limit of 1 eV.

(5) $$FWHM=a+b\sqrt{\left(E+cE^{2}\right)}$$

**Figure 2:**
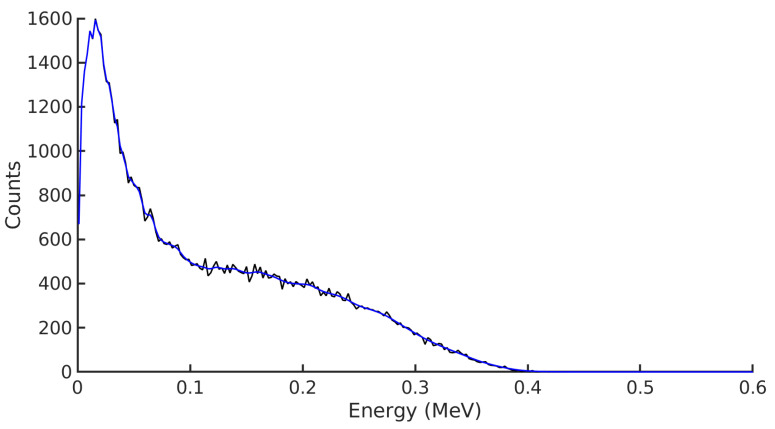
The simulated ${}^{90}$Sr spectrum acquired with the CdTe detector in the groundwater scenario, black, with the Gaussian broadening applied, blue.

A Geant4 simulation tracks radiation as it travels through matter and calculates the energy physically deposited in the material body of a radiation detector. However, the reality of radiation detection is more complicated. To make the simulated detector response more realistic, a Gaussian broadening was applied to the data. Manufacturer data were used to plot the correlation between the full width half-maximum (FWHM) and the gamma peaks of Am, ${}^{57}$Co,${}^{137}$Cs. The coefficients *a*, *b* and *c* were determined from Equation (5) and were used to apply a Gaussian broadening to the simulated spectra, as seen in Figure 2.

## 3. Results

The goal of this section is to demonstrate the deconvolution of a gross beta spectrum and evaluate the methodology to establish whether it can identify ${}^{90}$Sr activity in a contaminated groundwater environment. This technique is dependent on a priori knowledge of the radionuclides expected to be found in a groundwater borehole at a nuclear decommissioning site. If a radionuclide is not included in the response matrix, its presence cannot be determined. On the other hand, it is necessary to evaluate whether including many radionuclides in the response matrix can lead to false positives or otherwise distort the estimates of radionuclide activity. In Table 1, a borehole simulation is populated with ${}^{90}$Sr activity from 100,000 BqL${}^{-1}$ to 100 BqL${}^{-1}$. The response matrix, X, is populated with just ${}^{90}$Sr and ${}^{90}$Y initially, but ${}^{137}$Cs, ${}^{40}$K, and ${}^{235}$U are added in stages so that their effect on the estimated ratio of ${}^{90}$Sr and ${}^{90}$Y can be compared. This has demonstrated that the regression technique is effective at determining the ratio of ${}^{90}$Sr present in the groundwater down to activities as low as 100 BqL${}^{-1}$. The inclusion of the additional radionuclides into the response matrix does not produce any significant false positive values in the results and only alludes to a small deviation in the estimation of activity.

**Figure 3:**
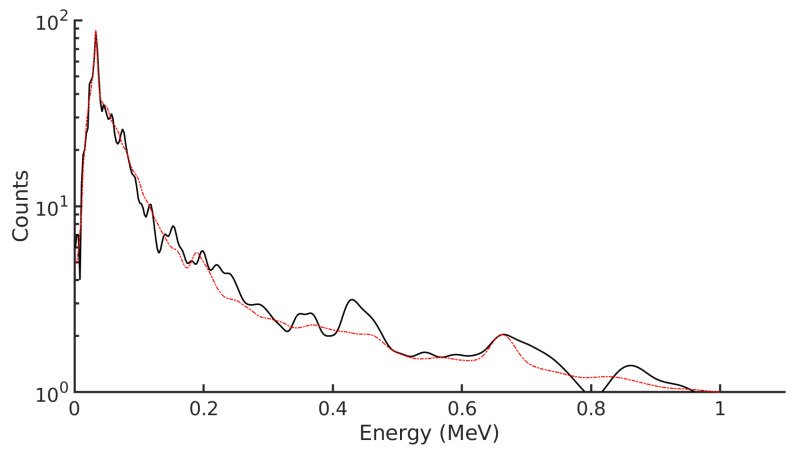
The spectrum corresponding to the radionuclide mix in Table 4 output by the detector is plotted in black, and the reconstructed spectrum resulting form the linear regression is plotted in red.

Table 2 demonstrates the ability to resolve ${}^{90}$Sr from ${}^{90}$Y decay at varying activity levels in groundwater. In reality, it is not well understood how these two radionuclides exist within the groundwater itself. The elements have different chemical properties, which means they interact with the environment differently; ${}^{90}$Sr and ${}^{90}$Y have different sorption rates in soil and move through the groundwater system at different rates. When a groundwater sample is collected, significant time passes between sampling and activity determination, which means that secular equilibrium is achieved at the point of counting. The spectrum of this mixture is plotted in Figure 3. Here, the measured spectrum is plotted in black. The gamma peak from ${}^{137}$Cs can be seen at 662 keV, with the X-ray photopeak protruding at the start of the spectrum. The long tail is due to beta emission from ${}^{90}$Y. Because ${}^{90}$Y decay results in more energetic particles, it produces a higher count-rate for the same activity when compared with ${}^{90}$Sr. This means that the ${}^{90}$Y spectrum is more well-defined for low activities, and this reflects the results shown in Table 2, where the ratio of ${}^{90}$Y is reproduced more reliably at low counts compared to ${}^{90}$Sr. However, it is still possible to identify the presence of ${}^{90}$Sr in strongly ${}^{90}$Y-contaminated groundwater. This scenario is unlikely to occur in reality, but serves as verification of the effectiveness of the technique.

${}^{90}$Sr and ${}^{90}$Y are not the only radionuclides found in contaminated groundwater at nuclear decommissioning sites; therefore, the deconvolution of a gross radiation spectrum may contain contributions from gamma emitters such as ${}^{137}$Cs and even alpha emitters such as ${}^{235}$U. The detector’s response to these radionuclides has been simulated and the deconvolution of ${}^{137}$Cs is presented in Table 3. ${}^{137}$Cs decay comprises beta decay up to an end-point energy of 0.512 MeV and gamma emission at 0.662 MeV. ${}^{137}$Cs is not typically found in high groundwater concentrations at Sellafield due to its strong sorption onto soil, with a maximum activity of 41.8 BqL${}^{-1}$. The results demonstrate the ability to deconvolve ${}^{137}$Cs activity from a mixture of high activity ${}^{90}$Sr and low activity ${}^{90}$Sr.

Finally, this method was applied to a realistic mixture of radionuclides that may be found in a groundwater borehole, as shown in Table 4. The results are reported with a 95% confidence interval. There is agreement between the simulated activity and the calculated activity as a result of the deconvolution of the gross spectrum. ${}^{235}$U was correctly not identified in the sample and the activities of ${}^{137}$Cs, ${}^{90}$Sr and ${}^{90}$Y were resolved despite the common overlap between their beta emission. The estimated activity of ${}^{90}$Sr 12.03 ± 0.56 kBq/L which leaves some discrepancy between the true and calculated activity. This is largely down to difficulties in resolving ${}^{90}$Sr and ${}^{90}$Y spectra. ${}^{90}$Y emission is more energetic and therefore less likely to be fully absorbed in the groundwater.

**Table 2:** The ratios of ${}^{90}$Sr and ${}^{90}$Y mixed to different activities as determined by linear regression analysis.

${}^{90}$Sr Activity BqL${}^{-1}$	${}^{90}$Y Activity BqL${}^{-1}$	${}^{90}$Sr Ratio	${}^{90}$Y Ratio
100,000	100,000	1.0000	1.0000
100,000	10,000	1.0390	0.0974
100,000	1000	1.0125	0.0011
100,000	100	0.9963	0.0003
100	100,000	0.0022	1.0000
100	10,000	0.00411	0.0973
100	1000	0.0015	0.0108
100	100	0.0000	0.0001

**Table 3:** The ratios of ${}^{90}$Sr and ${}^{137}$Cs mixed to different activities as determined by linear regression analysis.

${}^{90}$Sr Activity BqL${}^{-1}$	${}^{137}$Cs Activity BqL${}^{-1}$	${}^{90}$Sr Ratio	${}^{137}$Cs Ratio
100,000	100,000	1.0000	1.0000
100,000	10,000	0.9686	0.1004
100,000	1000	0.9810	0.0104
100,000	100	1.0072	0.0011
100	100,000	0.0022	1.0000
100	10,000	0.0000	0.1000
100	1000	0.0000	0.0103
100	100	0.0094	0.0011

**Table 4:** The estimate of ${}^{90}$Sr, ${}^{90}$Y and ${}^{137}$Cs activity from a realistic groundwater borehole.

Radionuclide	Simulated Activity (kBq/L)	Calculated Activity (kBq/L)
${}^{90}$Sr	10.00	12.29 ± 0.56
${}^{90}$Y	1.00	0.74 ± 0.05
${}^{137}$Cs	0.10	0.12 ± 0.004
${}^{235}$U	0	0.00

### 3.1. Alternative Detector

The previous sections describe the use of a 10 × 10 × 1 mm CdTe detector that was designed specifically for monitoring ${}^{90}$Sr contamination, but the results of applying this technique to other detectors’ configurations should be considered. A 1 mm detector is relatively unusual and detectors on the market are typically much thinner; indeed, thin solar cell panels may even be appropriated as radiation detectors. This section will consider the use of such detectors and evaluate whether they are viable for the linear regression technique. CdTe detectors of decreasing thickness were exposed to a simulated groundwater scenario containing 10 kBq/L of ${}^{90}$Sr, 1 kBq/L of ${}^{90}$Y and 0.1 kBq/L of ${}^{137}$Cs. The results of the simulations are displayed in Table 5. The 0.003 mm thick detector categorically produces the least agreement and it completely fails to estimate the activity of ${}^{90}$Sr in the groundwater. The material is not thick enough to sufficiently resolve in incident radiation upon its surface and as such produces highly compressed spectra. These spectra become impossible to distinguish from one another, which leads to the results seen in Table 5. Therefore, it is determined that in order to perform this technique, the thickness of the detector should fall within the range of 1 mm to 0.01 mm.

Of the detectors that fall into this range, it is notable that the detector with 0.1 mm thickness produces results that are closest to the true activities in the sample. This is somewhat paradoxical but can be explained by the effect of backscattering particles. Beta particles incident on a surface are prone to backscattering, where they only deposit a fraction of their energy and leave the detector before they are fully absorbed. This effect produces a peak in both the ${}^{90}$Sr and ${}^{90}$Y spectra collected with these detectors. When the detector is very thin, this peak is very pronounced. As the thickness of the detector increases, an increasing number of particles are distributed at higher channel numbers in the detector. This effect produces a second peak that is present in the 0.1 mm thick detector, but not in the 0.5 and 1 mm thick detectors. This characteristic of the spectrum in the 0.1 mm detector means that the regression can more easily resolve the ${}^{90}$Sr and ${}^{90}$Y spectra.

### 3.2. Impact of Suspended Solids

The groundwater found in monitoring boreholes commonly contains small concentrations of suspended solids, such as minerals and rock, which may remain from the drilling process or from inflow from the surrounding geological formation. To consider any potential influence of these particles on the measurement of radionuclides, these non-radionuclide components were added to the simulation based on analysis of measured particle concentrations, turbidity analysis and analysis of representative rock data.

The components considered include sodium, potassium, iron, calcium, magnesium, iron, aluminium, silicon and oxygen [28]. These components were distributed uniformly throughout the groundwater simulation, in accordance with the average and maximum monitored values. The results are reported in Table 6. When the groundwater contains the average concentration of all the components, 40 mg/L, the estimated activity of ${}^{90}$Sr is 10.09 ± 0.55 kBq/L, and returns a result of 9.74 ± 0.51 kBq/L for the maximum concentration at 300 mg/L. Therefore, it is not determined that these concentrations of components in the groundwater influenced the determination of the activity. The variation between the reported results is due to the random nature of radioactive decay and some inconsistency in the linear regression technique used.

Suspended solid concentrations can reach much higher levels than presented here; however, these can be considered to indicate a poorly designed or installed borehole or one requiring maintenance before a detector could be deployed.

### 3.3. Activity Determination by Energy Windows: Other Improvements

As seen in Table 4, there is room for improvement in the determination of ${}^{90}$Sr activity from a mixed source. One reason for this poor performance is the overlap of all the spectra up to the ${}^{90}$Sr end-point energy of 0.546 MeV. It has already been noted that the presence of an artificial peak in the ${}^{90}$Y spectrum can improve this deconvolution. As this peak is not present in detectors thicker than 0.5 mm, the estimation of the activities can be improved by performing a linear regression over two energy windows, low and high. The low energy window is classified as the energies up to 0.546 MeV and the high energy window contains all subsequent energies recorded in the detector. Therefore, no ${}^{90}$Sr decay is present in the high energy window and this window can be used to determine the activities of ${}^{90}$Y and ${}^{137}$Cs independently from ${}^{90}$Sr activity. The reconstructed ${}^{90}$Y and ${}^{137}$Cs spectra are then subtracted from the low energy window which allows the activity of ${}^{90}$Sr to be determined independently of higher-energy radionuclides. This technique was applied to a groundwater simulation containing 10.0 kBq/L ${}^{90}$Sr, 1.0 kBq/L of ${}^{90}$Y and 0.1 kBq/L of ${}^{137}$Cs and the results are displayed in Table 7. In this case, the estimated activity of ${}^{90}$Sr is erroneous by a margin of 0.77 kBq/L compared with 2.29 kBq/L. This demonstrates that performing a linear regression over a high energy and low energy window can improve the estimation of ${}^{90}$Sr activity in a mixed groundwater source.

The purpose of an in-situ detector is to collect results rapidly, and, as such, the previous spectra were collected over a time period of 5 h. Increasing this time period would result in spectra with a greater number of total counts. In Table 8, the results of determining 10 kBq/L activity over increasing time periods are presented. The results are consistent and it is not apparent that increasing the counting time is a factor in achieving estimates that are closer to the true ${}^{90}$Sr activity. Additionally, increasing the number of counts in the a priori matrix, X, makes no discernible difference to the estimated activity for the same 10 kBq/L ${}^{90}$Sr activity over a 5 h period.

## 4. Conclusions

The issue of quantifying ${}^{90}$Sr activity in groundwater boreholes with an in-situ detector has been presented and addressed by simulating the detector response and using a linear regression to estimate the activity of the radionuclides present. The response function of a CdTe detector to ${}^{90}$Sr, ${}^{90}$Y, ${}^{137}$Cs and ${}^{235}$U contamination in a groundwater borehole was simulated using the Monte Carlo software package Geant4. These response functions were used to create a database from which the detector’s response can be predicted. This method can be used to estimate ${}^{90}$Sr activity as low as 100 BqL${}^{-1}$, and it can be applied to identify ${}^{90}$Sr activity among a background of ${}^{90}$Y, ${}^{137}$Cs, ${}^{40}$K and ${}^{235}$U. It was shown that including additional radionuclides in the response function matrix does not significantly degrade the estimate of ${}^{90}$Sr activity. It was determined that detectors with thickness in the range of 0.1 to 1 mm can be used to apply this technique.

The issue of suspended solids in the groundwater was examined. The commonly found particles at Sellafield are sodium, potassium, iron, calcium, magnesium, iron, aluminium, silicon and oxygen, and these were included in the groundwater simulation at average and maximum concentrations. For these concentrations, the particles were not found to influence the results of radionuclide activity estimation.

One of the main impediments to beta spectroscopy is the overlapping of spectra that do not have characteristic features. This was resolved by applying the linear regression technique to two energy windows, determined by the end-point energy for ${}^{90}$Sr emission. Using this approach, the ${}^{90}$Sr activity in a 10.0 kBq/L ${}^{90}$Sr, 1.0 kBq/L ${}^{90}$Y and 0.1 kBq/L ${}^{137}$Cs mixed source was estimated as 10.77 ± 0.21 kBq/L.

These results have demonstrated how detector simulation and data regression can be used to enable in-situ semiconductor detectors to estimate ${}^{90}$Sr, ${}^{90}$Y, ${}^{137}$Cs and ${}^{235}$U activity in groundwater. However, some shortcomings are still present. Future research will investigate whether the application of different linear regression techniques can be used to improve the results. Some success may be found by incorporating different approaches to weighting to reduce the influence of outliers in the regression. The incorporation of other techniques, such as peak finding, may help to refine the results generated with this approach.

## Figures and Tables

**Table 1:** Results of analysis of a pure ${}^{90}$Sr mixture of decreasing activity with different radionuclides included in the detector response matrix.

${}^{90}$Sr Activity BqL${}^{-1}$	X [${}^{90}$Sr ${}^{90}$Y]	X [${}^{90}$Sr ${}^{90}$Y ${}^{137}$Cs]	[${}^{90}$Sr ${}^{90}$Y ${}^{137}$Cs ${}^{40}$K ${}^{235}$U]
100,000	[1.0000 0.0000]	[1.0000 0.0000 0.0000 ]	[1.0000 0.0000 0.0000 0.0000 0.0000]
10,000	[0.1017 0.0001 ]	[0.1010 0.0000 0.0000 ]	[0.1001 0.0001 0.0000 0.0000 0.0001]
1000	[0.0125 0.0000 ]	[0.0111 0.0000 0.0000 ]	[0.0114 0.0004 0.0000 0.0000 0.0002]
100	[0.0019 0.0000]	[0.0021 0.0000 0.0000 ]	[0.0021 0.0000 0.0000 0.0002 0.0000]

**Table 5:** The estimate of ${}^{90}$Sr, ${}^{90}$Y and ${}^{137}$Cs activity from a realistic groundwater borehole using a 3 µm GaAs detector.

Thickness (mm)	${}^{90}$Sr (kBq/L)	${}^{90}$Y (kBq/L)	${}^{137}$Cs(kBq/L)
1.0	12.03 ± 0.56	1.03 ± 0.14	0.12 ± 0.04
0.5	12.11 ± 0.52	0.99 ± 0.04	0.083 ± 0.003
0.1	10.42 ± 0.42	0.99 ± 0.02	0.099 ± 0.004
0.01	12.72 ± 0.91	0.93 ± 0.01	0.11 ± 0.013
0.003	0.00 ± 1.10	0.89 ± 0.01	5.81 ± 0.30

**Table 6:** The estimate of ${}^{90}$Sr activity from a realistic groundwater borehole with different concentrations of suspended non-radionuclide particles.

Total Concentration mg/L	Estimation of 10 kBq/L ${}^{90}$Sr Activity
Average 40	10.09 ± 0.55
Maximum 300	9.74 ± 0.51

**Table 7:** The estimate of ${}^{90}$Sr, ${}^{90}$Y and ${}^{137}$Cs activity from a realistic groundwater borehole.

Radionuclide	Simulated Activity (kBq/L)	Calculated Activity (kBq/L)
${}^{90}$Sr	10.00	10.77 ± 0.21
${}^{90}$Y	1.00	1.06 ± 0.03
${}^{137}$Cs	0.10	0.10 ± 0.01
${}^{235}$U	0	0.00

**Table 8:** The estimate of ${}^{90}$Sr activity for increasing lengths of measurement and therefore counts.

Length of Measurement (h)	Simulated Activity (kBq/L)	Calculated Activity (kBq/L)
5	10.00	10.05 ± 0.09
10	10.00	9.84 ± 0.03
24	10.00	9.69 ± 0.04

## Data Availability

Not applicable.

## References

[1] Cola A., Farella I. (2013). Electric Field and Current Transport Mechanisms in Schottky CdTe X-Ray Detectors under Perturbing Optical Radiation. Sensors.

[2] Egarievwe S.U., Roy U.N., Goree C.A., Harrison B.A., Jones J., James R.B. (2019). Ammonium Fluoride Passivation of CdZnTeSe Sensors for Applications in Nuclear Detection and Medical Imaging. Sensors.

[3] Turkington G., Gamage K.A.A., Graham J. (2018). Beta Detection of Strontium-90 and the Potential for Direct in Situ Beta Detection for Nuclear Decommissioning Applications. Nucl. Instrum. Methods Phys. Res. Sect. Accel. Spectrometers Detect. Assoc. Equip..

[4] Venara J., Ben Mosbah M., Mahé C., Astier J., Adera S., Cuozzo M., Goudeau V. (2020). Design and Development of a Portable *β*-Spectrometer for 90Sr Activity Measurements in Contaminated Matrices. Nucl. Instrum. Methods Phys. Res. Sect. Accel. Spectrometers Detect. Assoc. Equip..

[5] Nancekievill M., Espinosa J., Watson S., Lennox B., Jones A., Joyce M.J., Katakura J.I., Okumura K., Kamada S., Katoh M. (2019). Detection of Simulated Fukushima Daichii Fuel Debris Using a Remotely Operated Vehicle at the Naraha Test Facility. Sensors.

[6] Martin P.G., Moore J., Fardoulis J.S., Payton O.D., Scott T.B. (2016). Radiological Assessment on Interest Areas on the Sellafield Nuclear Site via Unmanned Aerial Vehicle. Remote. Sens..

[7] Kim J., Lim K.T., Ko K., Ko E., Cho G. (2020). Radioisotope Identification and Nonintrusive Depth Estimation of Localized Low-Level Radioactive Contaminants Using Bayesian Inference. Sensors.

[8] Ukaegbu I.K., Gamage K.A.A., Aspinall M.D. (2019). Nonintrusive Depth Estimation of Buried Radioactive Wastes Using Ground Penetrating Radar and a Gamma Ray Detector. Remote Sens..

[9] Alton T.T., Monk S.D., Cheneler D. (2017). Beta Particle Energy Spectra Shift Due to Self-Attenuation Effects in Environmental Sources. Nucl. Eng. Technol..

[10] Lee U., Choi W.N., Bae J.W., Kim H.R. (2019). Fundamental Approach to Development of Plastic Scintillator System for in Situ Groundwater Beta Monitoring. Nucl. Eng. Technol..

[11] Vajda N., Kim C.K. (2010). Determination of Radiostrontium Isotopes: A Review of Analytical Methodology. Appl. Radiat. Isot..

[12] Knoll G.F. (2000). Radiation Detection and Measurement.

[13] Kump P., Bai E.W., Chan K.S., Eichinger W. (2013). Detection of Shielded Radionuclides from Weak and Poorly Resolved Spectra Using Group Positive RIVAL. Radiat. Meas..

[14] White S.R., Wood K.T., Martin P.G., Connor D.T., Scott T.B., Megson-Smith D.A. (2021). Radioactive Source Localisation via Projective Linear Reconstruction. Sensors.

[15] Grujić S., Milošević M., Kozmidis-Luburić U., Bikit I. (2011). Monte Carlo Simulation of Beta Radiation Response Function for Semiconductor Si Detector. Nucl. Instrum. Methods Phys. Res. Sect. Accel. Spectrometers Detect. Assoc. Equip..

[16] Los Alamos National Laboratory: MCNP Home Page. https://mcnp.lanl.gov/.

[17] Lawson C.L., Hanson R.J. (1995). Solving Least Squares Problems.

[18] Bai E.W., Chan K.S., Eichinger W., Kump P. (2011). Detection of Radionuclides from Weak and Poorly Resolved Spectra Using Lasso and Subsampling Techniques. Radiat. Meas..

[19] Vajs I., Drajic D., Gligoric N., Radovanovic I., Popovic I. (2021). Developing Relative Humidity and Temperature Corrections for Low-Cost Sensors Using Machine Learning. Sensors.

[20] Venkatraman Jagatha J., Klausnitzer A., Chacón-Mateos M., Laquai B., Nieuwkoop E., van der Mark P., Vogt U., Schneider C. (2021). Calibration Method for Particulate Matter Low-Cost Sensors Used in Ambient Air Quality Monitoring and Research. Sensors.

[21] Jeon B., Kim J., Lee E., Moon M., Cho G. (2021). Pseudo-Gamma Spectroscopy Based on Plastic Scintillation Detectors Using Multitask Learning. Sensors.

[22] Czerwinski D., Gęca J., Kolano K. (2021). Machine Learning for Sensorless Temperature Estimation of a BLDC Motor. Sensors.

[23] Tomal A., Santos J., Costa P., Lopez Gonzales A., Poletti M. (2015). Monte Carlo Simulation of the Response Functions of CdTe Detectors to Be Applied in X-Ray Spectroscopy. Appl. Radiat. Isot..

[24] Neuer M.J. (2013). Spectral Identification of a 90Sr Source in the Presence of Masking Nuclides Using Maximum-Likelihood Deconvolution. Nucl. Instrum. Methods Phys. Res. Sect. Accel. Spectrometers Detect. Assoc. Equip..

[25] Green P.J. (1984). Iteratively Reweighted Least Squares for Maximum Likelihood Estimation, and Some Robust and Resistant Alternatives. J. R. Stat. Soc. Ser. Methodol..

[26] Agostinelli S., Allison J., Amako K., Apostolakis J., Araujo H., Arce P., Asai M., Axen D., Banerjee S., Barrand G. (2003). Geant4—A Simulation Toolkit. Nucl. Instrum. Methods Phys. Res. Sect. Accel. Spectrometers Detect. Assoc. Equip..

[27] Zhengming L. (1987). A Numerical Method for Solving the Fredholm Integral Equation of the First Kind and Its Application to Restore the Folded Radiation Spectrum. Nucl. Instrum. Methods Phys. Res. Sect. Accel. Spectrometers Detect. Assoc. Equip..

[28] (2009). Elemental Composition of [REDACTED] Sediments.

